# Low-host double MDA workflow for uncultured ASFV positive blood and serum sample sequencing

**DOI:** 10.3389/fvets.2022.936781

**Published:** 2022-09-20

**Authors:** Chengjun Zhang, Tangyu Cheng, Dongfan Li, Xuexiang Yu, Fangzhou Chen, Qigai He

**Affiliations:** ^1^State Key Laboratory of Agricultural Microbiology, Huazhong Agricultural University, Wuhan, China; ^2^College of Veterinary Medicine, Huazhong Agricultural University, Wuhan, China; ^3^The Cooperative Innovation Center for Sustainable Pig Production, Huazhong Agricultural University, Wuhan, China

**Keywords:** African swine fever, next generation sequencing (NGS), genome sequencing, nanopore sequencing, workflow

## Abstract

African swine fever (ASF) is a highly lethal and contagious disease caused by African swine fever virus (ASFV). Whole-genome sequencing of ASFV is necessary to study its mutation, recombination, and trace its transmission. Uncultured samples have a considerable amount of background DNA, which causes waste of sequencing throughput, storage space, and computing resources. Sequencing methods attempted for uncultured samples have various drawbacks. In this study, we improved C18 spacer MDA (Multiple Displacement Amplification)-combined host DNA exhaustion strategy to remove background DNA and fit NGS and TGS sequencing. Using this workflow, we successfully sequenced two uncultured ASFV positive samples. The results show that this method can significantly reduce the percentage of background DNA. We also developed software that can perform real-time base call and analyses in set intervals of ASFV TGS sequencing reads on a cloud server.

## Introduction

The African swine fever virus (ASFV) is the only member of the Asfarviridae family that causes contagious and lethal diseases. The first case of ASF was reported in Kenya in 1921 (1), while it was first documented in 2018 in China (2). Since then, 14 countries reported an ASFV outbreak between 2018 and 2021 in Southeast Asia (3). The disease has caused significant economic loss to the pig industry worldwide. So far, there have been no commercially effective vaccines, although inactivated, subunit, DNA, and gene-deleted vaccines that could offer at least partial protection against ASF exist (4, 5). A few gene-deletion vaccines and poxvirus vector-based vaccines have shown promising results in animals (6, 7), but further research is needed before their commercialization. Until now, ASFV has still been considered the most threatening virus for the pig industry.

ASFV is a double-stranded DNA virus of about 180–220-nm long. Its genome size is around 170–190 kb and encodes 150 proteins. Based on the 3'-term of the B464L gene, ASFV can be divided into 24 genotypes (8). However, previous studies have indicated that B464L is a relatively conserved gene. Research has been conducted to study different isolates, and it has been concluded that even those with the same genotype can have different phenotypes. A study showed several recombination possibilities between different strains (9). Experiments confirmed that some gene deletion viruses show widely varying virulence (6, 10, 11). Whole genome sequencing is considered necessary to better analyze this variation.

The first research on the ASFV genome using Sanger sequencing was published in 1995 (12). Nowadays, we can easily use high throughput sequencing platforms like MGI, Illumina, and Ion torrent to perform NGS sequencing. However, unlike the human or bacterial genome, the ASFV genome is small, and the uncultured clinical tissue and blood samples have a considerable amount of background DNA from the host, causing the ASFV reads to occupy between 0.01 and 0.1% of the total DNA (13, 14). Sequencing that can reach a hundred times the sequencing depth of the ASFV genome is usually needed to obtain a high-quality genome, implying that about 20–200 G of data is required to obtain the ASFV genome. This process, including the analysis and storage of data, can be very costly. Many attempts are being made to obtain the ASFV genome with low-cost techniques. Some experiments were conducted based on un-methylated DNA enrichment in sequencing. This implies removing the highly methylated host DNA (13, 15). Other trials were based on bait hybridization and used 11,958 biotinylated RNA-baits and Streptavidin beads to bait the DNA library that can hybridize with these baits (16). Long amplicon sequencing can also be applied, where only 40% of the region of the ASFV genome is focused on by using 6 pairs of primers to cover those regions (17). We compare these methods in Table 1 and conclude that researchers either faced difficulties with biased results, limited application, or the need to synthesize an extensive bait library.

**Table 1:** Sequencing strategy for an uncultured sample compared with LHDM.

	**Required sequencing data**	**Bias**	**Cost**	**Fit with third generation sequencing**
Directly sequencing	Very high	Low	High (sequencing cost)	Yes, but not recommended
Hybrid capture method	Low	Relatively high	High (library)	No
Long distance PCR method	Low	High(can get full genome)	Low	Contrived
Un-methylated DNA enrichment	Relatively high	Low	Relatively high (sequencing cost)	Yes
LHDM (workflow in this research)	Relatively low	Low	Low	Yes

Unlike the NGS sequencing method based on terminal end fluorescent-labeled dNTP and optical signal reader, TGS sequencing technology is developed on nanopore and electrical signal analysis. In this case, the sequencing equipment is smaller and can respond to real-time analysis. It can also generate longer reads and is not affected by GC bias. Long reads simplify the appraisal of deletions, insertions, translocations, and other genome-wide changes (18). They can also help cross high complex and repeated regions of the ASFV genome in the assembling stage (14). A study was conducted to sequence the ASFV genome using the Nanopore sequencing method and obtained 0.15% of the reads mapped to the ASFV genome (19). TGS needs high quality and a sufficient amount of long DNA to ensure it can successfully build the library and obtain satisfactory sequencing efficiency. Compared with NGS, which involves parallel sequencing of millions of reads, TGS has only 512 (the MinION and GridION flow cell) and 2,675 (the PromethION flow cell) nanopores to perform sequencing at the same time, implying that it is more sensitive to background DNA when sequencing an impure sample. Furthermore, the chip's life is highly affected by accumulated sequencing time and background DNA, indicating a need for longer sequencing time and higher cost.

After removing background DNA, the DNA concentration is usually <200 pg/ul, making it challenging to build the TGS library for sequencing. MDA (multiple displacement amplification) is a method for DNA amplification that is widely used in genome amplification. MDA is based on a DNA polymerase called phi29 found in the Bacillus subtilis phage. This DNA polymerase is low biased and, compared to other DNA polymerases, has advantageous features, such as high fidelity, strong strand displacement, and ongoing isothermal amplification. DNA can be amplified a thousand times in a typical overnight reaction, and DNA larger than 10 Kb can be yielded, making it optimum for TGS sequencing. However, MDA is unsuitable for ultra-low DNA input (<10 ng), which is the starting material obtained after treatment with our nuclease digestion strategy. This is due to the high percentage of junk DNA generated by template-independent amplification (20, 21). Researchers found that using a 5′ end block with a C18 spacer random primer can eliminate template-independent amplification (22). However, as the stand procedure of Oxford nanopore protocol requires the adaptor and barcode to ligate to 5′ ends of DNA, it is assumed that the 5′ end block with a spacer can avoid DNA ligation with adaptor and nanopore sequencing. Moreover, hyper-branched DNA, the final form of MDA product, will also block the nanopore, resulting in low sequencing efficiency and potential death of the nanopore. These issues need to be solved so that nanopores can be used to sequence ASFV samples.

**Figure 1:**
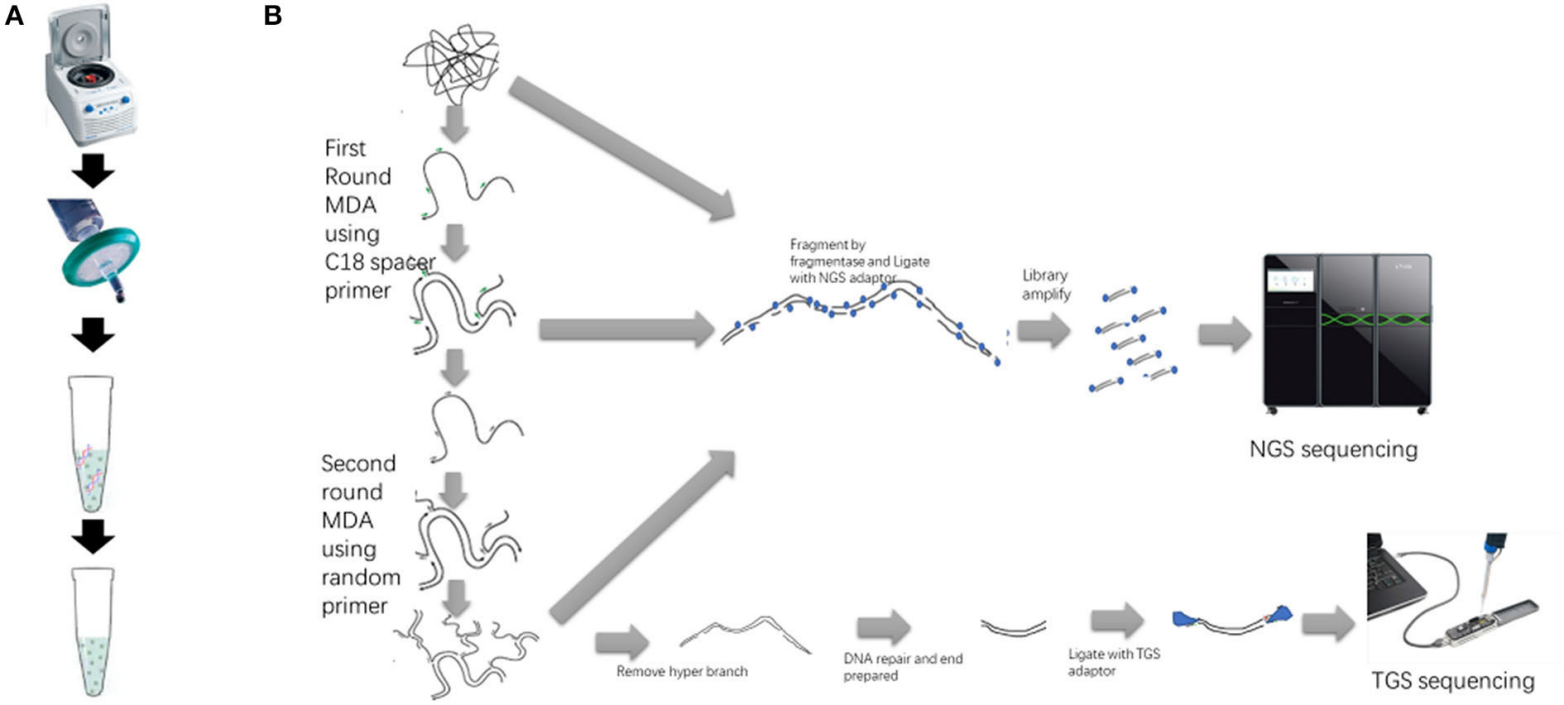
Sample preparation, double MDA, and library preparation workflow of NGS and TGS. **(A)** The sample preparation procedure includes centrifugation, filtration, and digestion with DNase mix. **(B)** The sequencing library preparation procedure. The first-round MDA product and the second-round MDA product can also be used to build the NGS library.

In this study, we combine nuclease enzyme mix digestion and double MDA to generate low host percentage and enough long DNA for NGS and TGS library building (Figure 1). We successfully used our LHDM workflow to sequence two uncultured ASFV positive samples. Because of the significant reduction of background DNA percentage, we were able to obtain the genome of ASFV from the uncultured sample at low costs in the sequencing throughput, smaller storage space, and computing resources. TGS can provide an early estimate of samples before NGS and help in the assembly because of its flexibility and its ability to sequence long reads. Using the LHDM TGS software we developed, real-time analysis can be done for ASFV TGS sequencing on the cloud server.

## Materials and methods

### Sample collection and preparation

Blood samples were collected from suspected ASF-infected pigs, Sample 1 being a serum sample and Sample 2 an anti-agglutinated blood sample. Two cycles of freezing and thawing were carried out to induce cell rupture. The samples were centrifuged at 8,000 × g for 10 min and then filtered using a 0.45-μm filter. A 100-μl sample was diluted to 200 μl to obtain a solution of 15-mM Tris-HCL, 30-mM MgCl_2_, 2 × anti-anti (Thermofisher15240062). Approximately, 11 μl of enzyme mix [5-μl DNase I (Takara 2270A), 5-ul Turbo DNAse (ThermofisherAM2239), 1-μl ultranuclease (Hzymes HBP000107)] were added, and the sample was placed in an incubator at 37°C for 1 h. After adding 15-ul 0.5-M EDTA, DNases were inactivated by Qiagen buffer AL and Proteinase K at 60°C for 20 min, and the DNA was extracted using the Qiagen DNA mini kit (51304).

### DMDA amplification

Approximately 10.6-μl DNA and a 5-μl 100-μM 5'end block with a C18 spacer random primer were denaturalized at 95°C for 5 min. Approximately, a 2-μl 10 × Phi29DNA polymerase buffer, 0.4-μl BSA, 1-μl 10-mM DNTP, and 10-U Phi29DNA polymerase were added to each sample, and then incubated under 28°C for 15 h. The sample was purified using the Monarch PCR & DNA cleanup kit (NEB T1030S). A volume of 10.6-μl purified DNA, and a 5-μl 100-μM random primer were used, and the first round of reaction repeated at an incubation temperature of 30°C for 6 h. The Equalbit 1 × dsDNA HS Assay Kit (Vazyme EQ121-011) was used to qualify DNA concentration. Approximately, 10-U S1 Nuclease (Takara 2410A) for 1-ug double-strand DNA was added to the DNA to remove hyper-branched parts of DNA. After digestion at 23°C for 20 min, the sample was purified using the Monarch PCR & DNA cleanup kit (NEB T1030S).

### TGS library preparation

A TGS library was prepared using the Oxford nanopore technology Native barcoding amplicons (with EXP-NBD104, EXP-NBD114, and SQK-LSK109) protocol. Approximately, 600-ng DNA was used as starting material. The Monarch PCR & DNA cleanup kit (NEB T1030S) was used to purify the samples rather than AMPure XP beads after the DNA repair and end preparing step. In the last step, the beads were washed using SFB (Short Fragment Buffer).

### NGS library preparation

An NGS library was constructed using VAHTS Universal Plus DNA Library Prep Kit (Vazyme, NDM617). Approximately, 10-ul DNA was added to FEA reaction and incubated at 37°C for 17 min for fragmentation. Approximately, 1:20 diluted DNA Adapters for MGI (vazyme, NM108) was used in Adapter Ligation. Approximately, 17 PCR cycles were used in the library amplification stage. Size selection was then carried out using 68 ul in the first round and 20 ul in the second round. For samples not treated by DNase mix, 1:10- and 1:2-diluted DNA Adapters were used, separately.

### TGS sequencing

Sequencing was performed on an R9.4.1 flowcell with a Minion device (Oxford nanopore technology). The Minokow software was used to collect the raw signal on a laptop. Approximately, 4,000 reads were set for a fast5 file. Basecalling in Minokow was disabled. Using our software, raw fast5 files were uploaded to a cloud computer with 2080ti GPU to perform real-time basecall and coverage analysis.

## Results

### NGS sequencing result

NGS sequencing was performed on the MGI T7 platform. Approximately, 15.17-M and 31.94-M reads from Samples 1 and 2 were obtained, respectively. After removing adaptors and the low-quality base, Sample 1 maintained 13.66-M high-quality reads, and Sample 2 maintained 29.36-M high-quality reads. By mapping to reference, both paired reads were considered as Mapped reads. Approximately, 1.4% of reads from Sample 1 were mapped to the ASFV genome, and Sample 2 had 44.9% reads mapped to the ASFV genome. Compared with the sample not treated by DNase mix, the ASFV reads had been raised 46 and 112 times, respectively. Detailed results are shown in Table 2.

**Table 2:** NGS sequencing data information and the mapping rate to ASFV genome.

	**Raw reads**	**Raw base**	**Reads after filter by fastp**	**Base After filter by fastp**	**Q30 base after filter**	**Mapped readsand mapping rate to ASFV genome**
Sample 1-untreated	30.93 M	4.63 G	30.91 M	4.51 G	4.32 G	11.25 K (0.03%)
Sample 1	15.17 M	2.28 G	13.66 M	1.94 G	1.73 G	0.19 M (1.4%)
Sample 2-untreated	37.89 M	5.68 G	37.87 M	5.46 G	5.16 G	157.60 K (0.4%)
Sample 2	31.94 M	4.79 G	29.36 M	4.12 G	3.65 G	13.20 M (44.9%)

### TGS sequencing result

**Figure 2:**
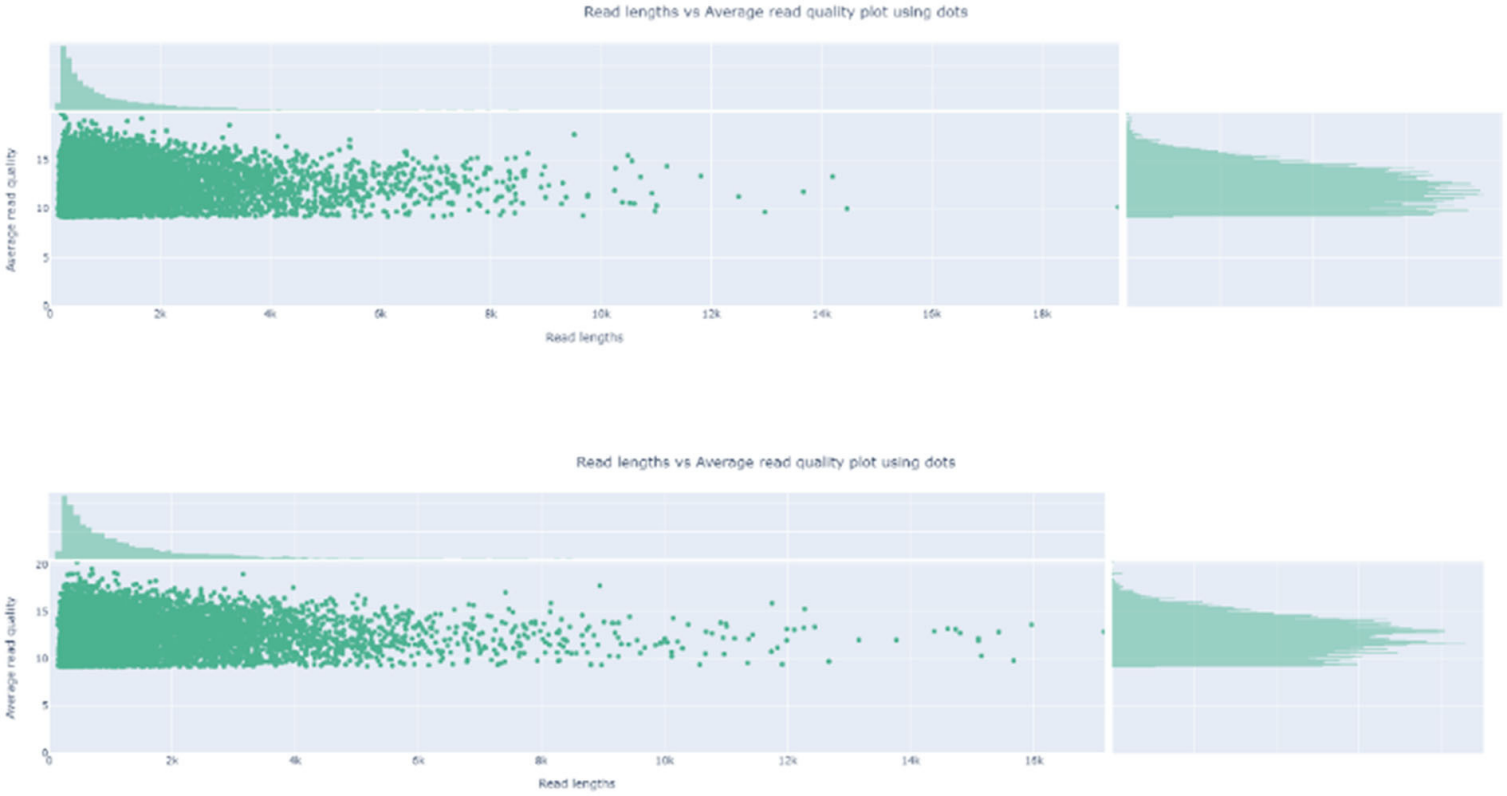
Read Length and the average read quality plot. The read length and quality were visualized by nanoplot.

After 8-h run, sequencing was stopped, and the R9.4 flow cell was washed. Base-calling and barcoding were performed. A total of 1.97-G data and 1.5-M reads were obtained. The longest read was 325,663 bp, which was not ASFV nuclear acid. After mapping to the Pig/HLJ/2018 strain, the mapping rate was 3.62 and 0.02%. Details of sequencing are shown in Table 3. Read length and quality are shown in Figure 2.

**Table 3:** TGS sequencing data information and the mapping rate to ASFV genome.

	**Reads**	**Reads N50**	**Longest read length**	**Total base**	**Reads mapped rate to ASFV genome**	**Mapped reads N50**	**Longest read length**
Sample 1	658,546	2,264	325,663	0.90 G	126 (0.02%)	4,113	12,714
Sample 2	925,609	2,175	30,327	1.17 G	33,482 (3.62%)	2,464	27,106

### Real-time analysis by TGS

**Figure 3:**
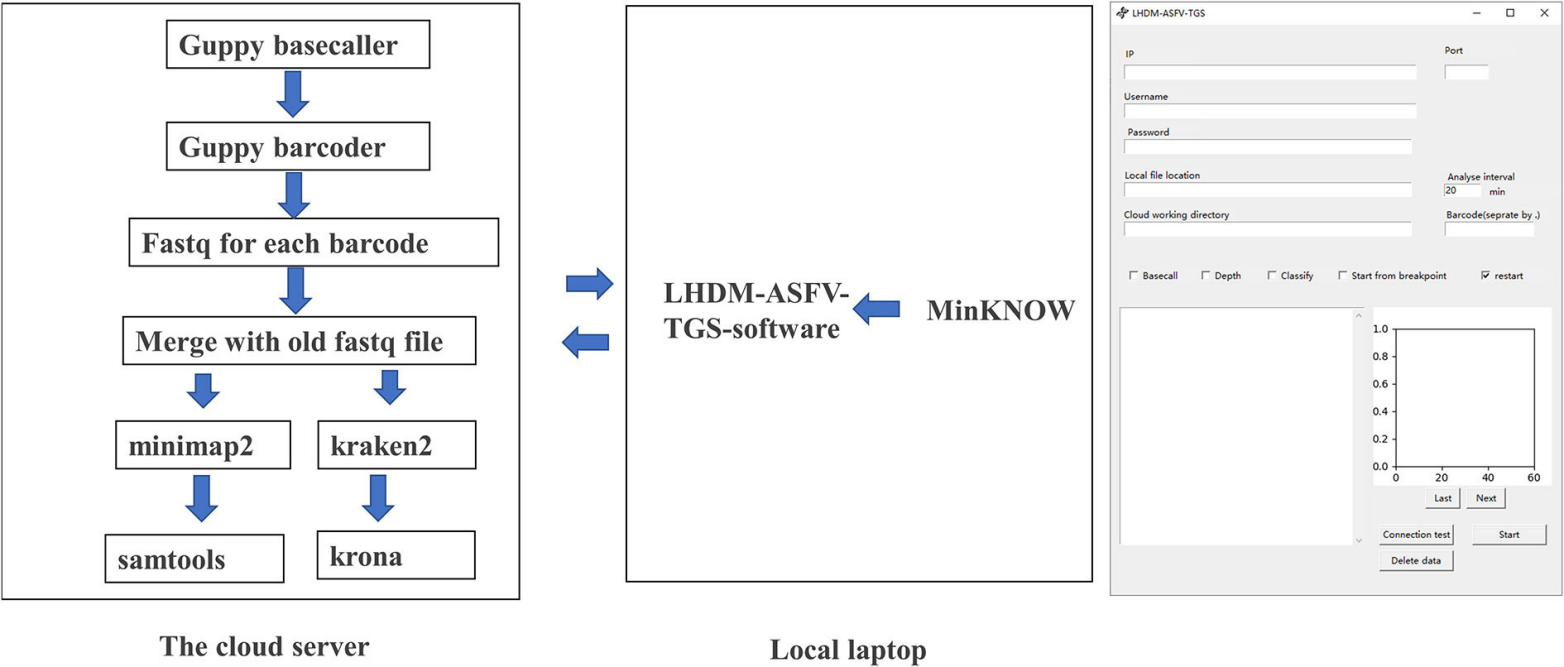
The analysis dataflow and the interface of LHDM-ASFV-TGS software.

**Figure 4:**
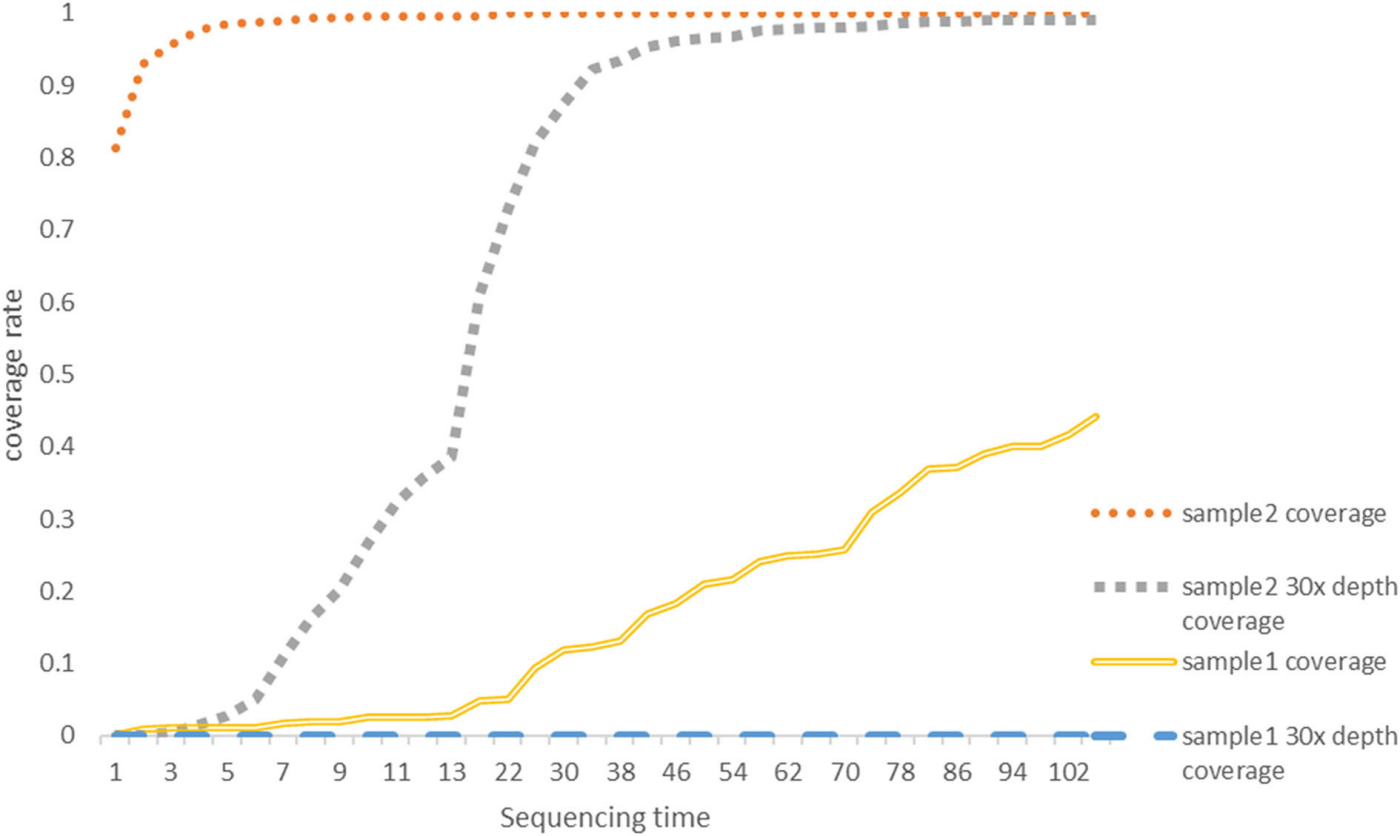
The genome coverage rate with a sequencing time graph. The coverage data were generated by LHDM-ASFV-TGS software. The units of the X axis are 5 min; the vertical axis represents the coverage rate; 30-x depth means for a specific site in the genome of ASFV was sequencing at least 30 times. Each curve represents the coverage and the 30-x coverage rate of Sample 1 and Sample 2 on the graph.

Our set in Minokow was generating one fast5 for every 4,000 reads. Our software (Figure 3) was set to upload new files to cloud GPU in the 1st hour after 5 new fast5 had been generated. This takes about 5 min. Based on real-time analysis by our software, the first ASFV reads were detected for Sample 2 and Sample 1 after 5 and 10 min, respectively. Approximately, 30-x depth is the requirement for the assembly in most assembled software. Genome coverage of Sample 2 reached 81% within the first 5 min, 92% within 10 min, and 99% within 40 min. Position with 30-x depth reached 90% in 2.5 h and 99% in 7.7 h. Detailed results are shown in Figure 4 below. The dataflow and the interface of LHDM-ASFV-TGS software are shown in Figure 3.

### Metagenomic analysis of NGS and TGS data

**Figure 5:**
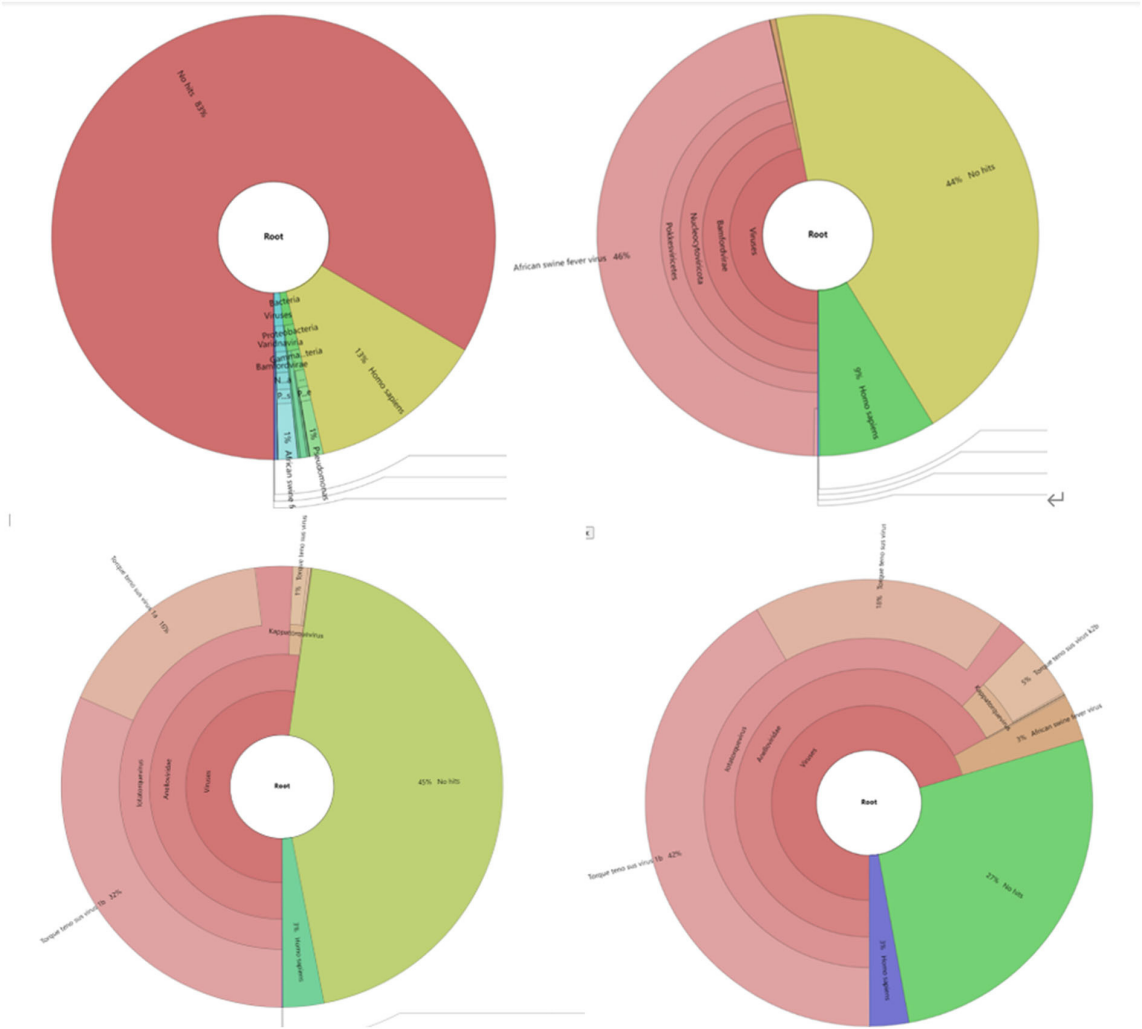
Metagenomic analysis of sequencing data with the Minisub database and kraken2. Different species are shown in different colors. (1) Metagenomic classification results of Sample1 NGS sequencing results; results for ASFV shown in blue. (2) Metagenomic classification results of Sample 1 NGS sequencing results, results for ASFV shown in pink. (3) Metagenomic classification results of Sample 1 TGS sequencing results, results for ASFV not shown due to low-percentage reads. (4) Metagenomic classification results of Sample 2 TGS sequencing results, results for ASFV shown in light brown color.

Because of the low-mapping rate of Sample 1, and reads mapped ratio difference between TGS and NGS, a metagenomic analysis was performed using Kraken2 and the minusb database, which contains all the virus genomes. Results (Figure 5) showed that Torque teno sus virus (42% in Sample 1 and 53% in Sample 2), a single-stranded circular DNA virus, occupied most reads in the TGS result.

There was no Sus Scrofa genome in the kraken2 database. Hence, bowtie2 was used to map unclassified reads to the sus scrofa genome. The mapping rates were 61.84 and 72.37% for Sample 1 and Sample 2, respectively, implying that most of the unhit reads belonged to unknown species in our sample.

### Assembled genome information

Considering that not all the background DNA belongs to pig and that a high percentage of reads belonged to unknown species, we chose to map all the reads to the ASFV genome before assembly. Because of the high depth, NGS Assembly was relatively easy. TGS reads of Sample 1 were insufficient for assembly. Hence, no TGS contig of Sample 1 was generated. Assembly was done by canu and Flye, respectively. NGS and TGS contigs were connected by ragtag and hand. Medak polished TGS scaffold. The final genomes of Sample 1-NGS, Sample 2-NGS, and Sample 2-TGS were 187,984, 188,050, and 186,575, respectively.

### Genome phylogenetic and mutation analysis

**Figure 6:**
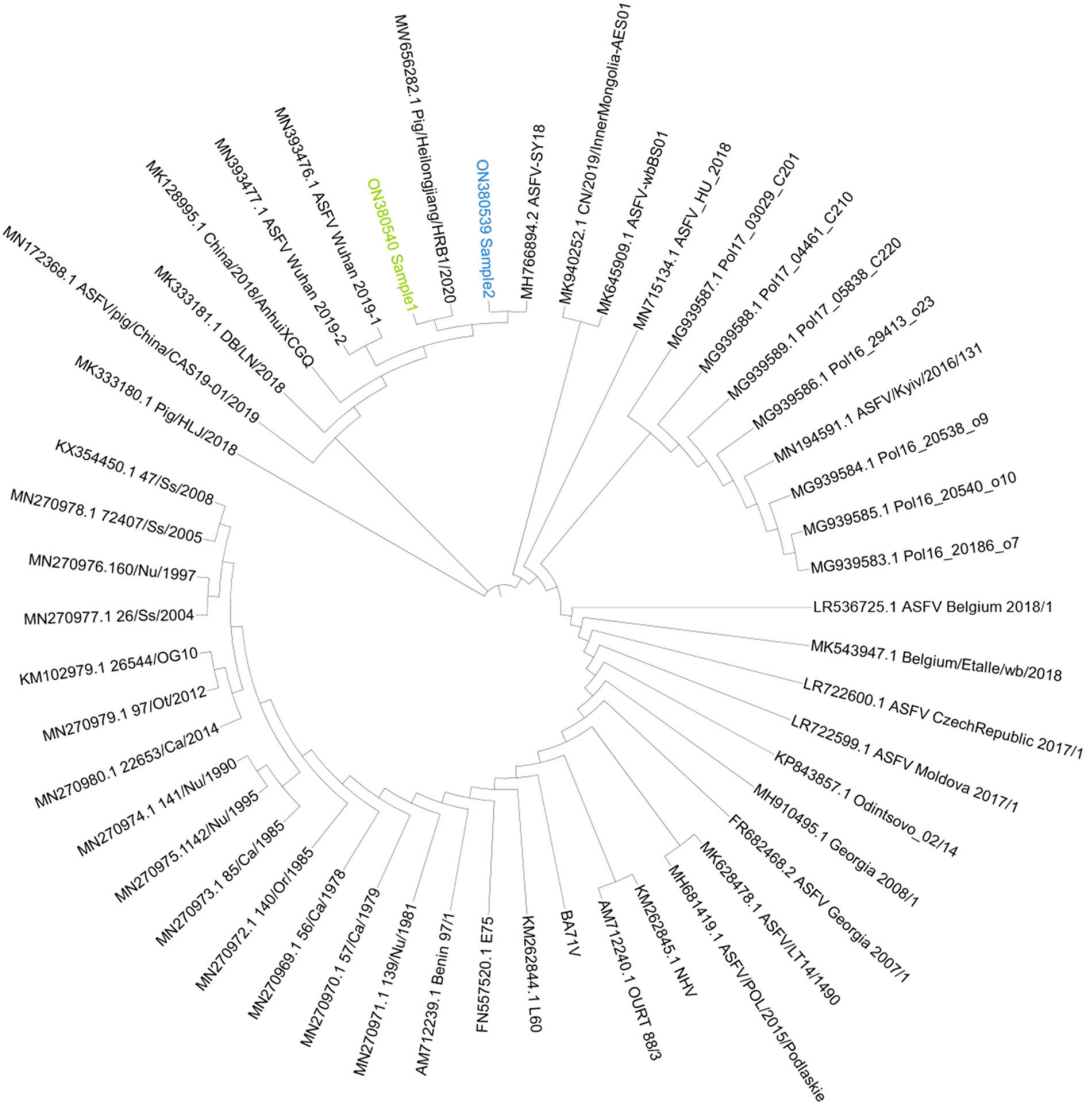
The genome phylogenetic tree. The phylogenetic tree was constructed by iqtree and visualized by ITOL. Sample 1 was marked as green, and Sample 2 was marked as blue.

Based on the genome phylogenetic tree in Figure 6, it was deduced that the two isolates of Sample 1 and Sample 2 were closest to ASFV-SY18 and HRB/2020 isolates, respectively. The two isolates might be the Offspring of the first reported isolate PIGHLJ/2018 in China. Full genomes offer more information for virus evolution and traceability.

Compared to PIG/HLJ/2018, Sample 1 had 5 single sites in genes MGF_110-3L, MGF_360-6L, A240L and C475L, and Sample 2 had 8 single-site mutations in genes MGF_300-1L, MGF_505-2R, MGF360-15R, F1055L, CP530R, NP1450L, and I215L. Sample 1 had 3 single nucleic acid deletions in genes MGF 300-2L, MGF 110-7L, and MGF 110-14L and 1 partial deletion in gene MGF 110-114L. Sample 2 had 1 single site deletion in gene I11L, 2 single-site insertions in gene MGF110-7L and gene D1133L, and 2 partial deletions in gene ASFV_G_ACD_00350 and MGF_110-13L. There was no gene-wide gene deletion in the two isolates. Detailed information can be found in Supplementary Tables S1, S2.

### Comparison between TGS assemble and NGS assemble

Compared to the NGS assemble, the TGS assemble has 110 indel sites, out of which 14 were insertion sites, all being in the poly region. Approximately, 96 nucleotides were deleted, out of which 44 were from the poly region, 1 from the non-poly region, and 51 from the segment deletion. There is no SNP site when comparing TGS with the NGS assemble. All the indel information can be found in Supplementary Table S3.

## Discussion

It is possible to obtain low-host DNA with a high percentage of the ASFV genome using our enzyme mix and buffer settings. ASFV occupied 1.4 and 44% of the total reads in the two NGS samples, which is a significant improvement compared to the untreated sample. However, the elimination of background DNA depends on digesting cell-free nucleic acids and requires complete unbroken virion. The DNA of inactivate virus can also be digested, depending on how the virus was inactivated. This workflow needs a sample with a lived virus, implying that it needs to be processed in an ABSL-3 laboratory and might limit its usage.

It was observed that the concentration of Mg^2+^ in the buffer depended on the common sample type. Mg^2+^ is also critical to enzymatic activity. Since most blood samples were collected using the EDTA anticoagulation tube, a reaction buffer with a final concentration of 30-mM Mg^2+^ and 15 mM of Tris-HCL was used in this study. NGS data show that the ASFV DNA percentage was at the same level as the host, showing that the enzyme was used at its optimum efficiency in our experiment.

It is also important to consider that this workflow sequences all DNA viruses, including bacteriophages. If the sample was contaminated, bacteria and bacteriophages could replicate very fast. Phages cannot be removed using this workflow, so it is crucial to ensure that an unspoiled sample is used.

TGS can generate very long reads, and we obtained the longest read of 27 Kb, covering 14% of the ASFV genome. The long reads can help us easily cross repeated regions when assembling and quickly find the indel gene.

The TGS sequencing base call requires a high-performance GPU. Most laptops might not be able to carry out this process effectively due to the need for long hours of continuous work, increasing the risk of overheating. This partly limits the usage of nanopore technology. In this study, we wrote the script to automatically upload the sequencing data and analyses to other Linux GPU servers, which is the most accessible solution to this issue.

More than 24–30 h was needed to finalize the NGS PE150 sequencing, especially for the sequencing of a low host sample that only needs a very low output yield. In this case, a large amount of samples is necessary for one run. TGS sequencing can be stopped at any time, and the chip reused, making the sequencing process more flexible. Our software can help decide when to stop sequencing. Sample 1 of TGS showed a low percentage of ASFV reads. We did not keep sequencing to get enough ASFV depth. At this stage, sequencing follows the Bernoulli process. Its sensitivity depends on the target-sample background ratio. Only increasing the sequencing output has a limited effect (23). Our software can be downloaded on the following link https://github.com/chauncyzhang/LHdMDAsoftware.

Running the NGS equipment whenever needed is neither practical nor cost-effective in most cases and laboratories. It usually takes more than 3 weeks to get NGS data from commercial companies on the market, especially considering the low data requirements for each sample. We can use TGS to decide whether the sample should be sequenced and how much data are suitable for the sample.

Because the DNA input is usually lower than all commercial kit ranges, it may fail when building the NGS library. We successfully built the NGS library using our protocol because a junk DNA phenomenon is prevented by the C18 spacer random primer. The output of Step 1 MDA or Step 2 can also be used as the starting material to build the NGS library.

According to our TGS results, the torque teno sus virus (42% reads in Sample 1 and 53% reads in Sample 2) has a single-stranded circular DNA, occupying a considerable percentage of total reads. Single-stranded circular DNA cannot be built into the NGS library using our method but can be amplified by the TGS workflow. Meanwhile, all double-strand DNA can be built into the TGS Library, the same as NGS. Because of high-background DNA and amplification of ssDNA and circle DNA, the sequencing results are unacceptable, so we did not perform TGS on the untreated sample by DNAse mix. Considering the high positive rate of this virus in pigs (40–100%) (24, 25), AMPure XP Beads or high molecular weight DNA extract kits (e.g., Qiagen 12462 or NEB T3060L) can be used to remove the TTSV DNA from the virus for further experimentation. These kits can assist in the removal of small circular DNA and increase the input DNA length.

Growth of ASFV in PAM cells and ultracentrifuge can also be used to obtain low-host high amount of ASFV DNA but requires the use of an ABSL-3 lab for virus isolation and passage. However, there is no ultracentrifuge equipment in some ABSL-3 laboratories. It has been reported that ASFV may lose some genes in the MGF region in the passage in 293T cells (26). Nevertheless, according to our results, there are only a few mutations in these two isolates, and other isolates sequenced by some research also only have a few mutations (19, 27). Considering that ASFV may mutate during the passage, it might be a better strategy to carry out sequencing before virus isolation.

Compared to the NGS assemble, we found that most false sequencing occurs in repeat poly regions by TGS, which is one of the drawbacks of TGS. We still need NGS data to polish the TGS genome at this time, but the quality of the TGS assemble is high and can also provide a certain amount of information. With the release of the R10 flow cell and Q20+ reagent, it is possible to obtain higher-quality TGS sequencing data.

## Data availability statement

The datasets presented in this study can be found in online repositories. The names of the repository/repositories and accession number(s) can be found below: https://www.ncbi.nlm.nih.gov/genbank/, ON380539; https://www.ncbi.nlm.nih.gov/genbank/, ON380540.

## Author contributions

CZ, FC, and QH contributed to the conception or design of the work and drafted the manuscript. CZ and TC completed the most experiments and data analysis. DL and XY had contribution on sample obtain process and other experiments. QH managed the whole project. FC and QH applied the fundings. All authors have reviewed and edited the manuscript, contributed to the article, and approved the submitted version.

## Funding

This project was funded by the Epidemiological Characteristics and Evolution of African Swine Fever Virus and Early Detection Technology (31941004), Young Scientists Fund of the National Natural Science Foundation of China (Grant No. 31902300), and the Research and Application of Key Technology Against African Swine Fever (2019ABA089).

## Conflict of interest

The authors declare that the research was conducted in the absence of any commercial or financial relationships that could be construed as a potential conflict of interest.

## Publisher's note

All claims expressed in this article are solely those of the authors and do not necessarily represent those of their affiliated organizations, or those of the publisher, the editors and the reviewers. Any product that may be evaluated in this article, or claim that may be made by its manufacturer, is not guaranteed or endorsed by the publisher.
