# Supplementary material for: Low-host double MDA workflow for uncultured ASFV positive blood and serum sample sequencing
Source: Front Vet Sci. 2022 Sep 20;9:936781. doi: 10.3389/fvets.2022.936781 (PMC9531595; doi:10.3389/fvets.2022.936781)
Supplement: Supplementary file 1 [file Data_Sheet_1.DOCX]

| Gene name | gene locs in direct strand or complementary strand | gene start location | gene end location | site location in MK333180.1 | Nucleotides in MK333180.1 | Nucleotides in Genome of this strain |
| --- | --- | --- | --- | --- | --- | --- |
| MGF_110-7L | c | 9356 | 9769 | 9660 | - | g |
| MGF_110-14L | c | 13263 | 13613 | 13266 | c | - |
| MGF_110-14L | c | 13263 | 13613 | 13267 | c | - |
| MGF_110-14L | c | 13263 | 13613 | 13268 | c | - |
| MGF_110-13L | c | 14248 | 15072 | 14708 | c | - |
| MGF_110-13L | c | 14248 | 15072 | 14709 | c | - |
| MGF_110-13L | c | 14248 | 15072 | 14710 | c | - |
| MGF_110-13L | c | 14248 | 15072 | 14711 | c | - |
| MGF_110-13L | c | 14248 | 15072 | 14712 | c | - |
| MGF_110-13L | c | 14248 | 15072 | 14713 | c | - |
| in non-coding regions | in non-coding regions | in non-coding regions | in non-coding regions | 16664 | - | g |
| in non-coding regions | in non-coding regions | in non-coding regions | in non-coding regions | 16877 | g | - |
| ASFV_G_ACD_00350 | d | 19002 | 19136 | 19032 | g | - |
| ASFV_G_ACD_00350 | d | 19002 | 19136 | 19033 | g | - |
| ASFV_G_ACD_00350 | d | 19002 | 19136 | 19034 | g | - |
| ASFV_G_ACD_00350 | d | 19002 | 19136 | 19035 | g | - |
| ASFV_G_ACD_00350 | d | 19002 | 19136 | 19036 | g | - |
| ASFV_G_ACD_00350 | d | 19002 | 19136 | 19037 | g | - |
| MGF_300-1L | c | 19742 | 20548 | 20293 | c | t |
| MGF_300-1L | c | 19742 | 20548 | 20381 | g | a |
| in non-coding regions | in non-coding regions | in non-coding regions | in non-coding regions | 20835 | g | - |
| MGF_505-2R | d | 33133 | 34713 | 34309 | c | t |
| in non-coding regions | in non-coding regions | in non-coding regions | in non-coding regions | 44279 | t | - |
| in non-coding regions | in non-coding regions | in non-coding regions | in non-coding regions | 44280 | t | - |
| in non-coding regions | in non-coding regions | in non-coding regions | in non-coding regions | 44281 | t | - |
| in non-coding regions | in non-coding regions | in non-coding regions | in non-coding regions | 44282 | a | - |
| in non-coding regions | in non-coding regions | in non-coding regions | in non-coding regions | 44283 | g | - |
| in non-coding regions | in non-coding regions | in non-coding regions | in non-coding regions | 44284 | t | - |
| in non-coding regions | in non-coding regions | in non-coding regions | in non-coding regions | 44285 | t | - |
| in non-coding regions | in non-coding regions | in non-coding regions | in non-coding regions | 44286 | a | - |
| in non-coding regions | in non-coding regions | in non-coding regions | in non-coding regions | 44287 | a | - |
| in non-coding regions | in non-coding regions | in non-coding regions | in non-coding regions | 44288 | g | - |
| in non-coding regions | in non-coding regions | in non-coding regions | in non-coding regions | 44289 | a | - |
| in non-coding regions | in non-coding regions | in non-coding regions | in non-coding regions | 44290 | t | - |
| in non-coding regions | in non-coding regions | in non-coding regions | in non-coding regions | 44291 | a | - |
| in non-coding regions | in non-coding regions | in non-coding regions | in non-coding regions | 44292 | g | - |
| in non-coding regions | in non-coding regions | in non-coding regions | in non-coding regions | 44293 | t | - |
| in non-coding regions | in non-coding regions | in non-coding regions | in non-coding regions | 44294 | a | - |
| in non-coding regions | in non-coding regions | in non-coding regions | in non-coding regions | 44295 | g | - |
| in non-coding regions | in non-coding regions | in non-coding regions | in non-coding regions | 44444 | a | - |
| in non-coding regions | in non-coding regions | in non-coding regions | in non-coding regions | 44445 | t | - |
| in non-coding regions | in non-coding regions | in non-coding regions | in non-coding regions | 44446 | a | - |
| in non-coding regions | in non-coding regions | in non-coding regions | in non-coding regions | 44447 | g | - |
| in non-coding regions | in non-coding regions | in non-coding regions | in non-coding regions | 44448 | t | - |
| in non-coding regions | in non-coding regions | in non-coding regions | in non-coding regions | 44449 | t | - |
| in non-coding regions | in non-coding regions | in non-coding regions | in non-coding regions | 44450 | t | - |
| in non-coding regions | in non-coding regions | in non-coding regions | in non-coding regions | 44451 | a | - |
| in non-coding regions | in non-coding regions | in non-coding regions | in non-coding regions | 44452 | g | - |
| in non-coding regions | in non-coding regions | in non-coding regions | in non-coding regions | 44453 | t | - |
| in non-coding regions | in non-coding regions | in non-coding regions | in non-coding regions | 44454 | t | - |
| in non-coding regions | in non-coding regions | in non-coding regions | in non-coding regions | 44455 | a | - |
| in non-coding regions | in non-coding regions | in non-coding regions | in non-coding regions | 44456 | a | - |
| in non-coding regions | in non-coding regions | in non-coding regions | in non-coding regions | 44457 | g | - |
| in non-coding regions | in non-coding regions | in non-coding regions | in non-coding regions | 44458 | t | - |
| in non-coding regions | in non-coding regions | in non-coding regions | in non-coding regions | 44459 | c | - |
| in non-coding regions | in non-coding regions | in non-coding regions | in non-coding regions | 44460 | a | - |
| in non-coding regions | in non-coding regions | in non-coding regions | in non-coding regions | 44461 | a | - |
| in non-coding regions | in non-coding regions | in non-coding regions | in non-coding regions | 44462 | t | - |
| in non-coding regions | in non-coding regions | in non-coding regions | in non-coding regions | 44463 | a | - |
| in non-coding regions | in non-coding regions | in non-coding regions | in non-coding regions | 44464 | g | - |
| in non-coding regions | in non-coding regions | in non-coding regions | in non-coding regions | 44465 | t | - |
| in non-coding regions | in non-coding regions | in non-coding regions | in non-coding regions | 44466 | t | - |
| in non-coding regions | in non-coding regions | in non-coding regions | in non-coding regions | 44467 | t | - |
| in non-coding regions | in non-coding regions | in non-coding regions | in non-coding regions | 44468 | a | - |
| in non-coding regions | in non-coding regions | in non-coding regions | in non-coding regions | 44469 | g | - |
| in non-coding regions | in non-coding regions | in non-coding regions | in non-coding regions | 44470 | t | - |
| in non-coding regions | in non-coding regions | in non-coding regions | in non-coding regions | 44471 | t | - |
| in non-coding regions | in non-coding regions | in non-coding regions | in non-coding regions | 44472 | a | - |
| in non-coding regions | in non-coding regions | in non-coding regions | in non-coding regions | 44473 | a | - |
| in non-coding regions | in non-coding regions | in non-coding regions | in non-coding regions | 44474 | g | - |
| in non-coding regions | in non-coding regions | in non-coding regions | in non-coding regions | 44475 | t | - |
| in non-coding regions | in non-coding regions | in non-coding regions | in non-coding regions | 44476 | c | - |
| in non-coding regions | in non-coding regions | in non-coding regions | in non-coding regions | 44477 | a | - |
| MGF_360-15R | d | 49425 | 50255 | 50064 | g | a |
| F1055L | c | 59874 | 63020 | 61056 | g | a |
| in non-coding regions | in non-coding regions | in non-coding regions | in non-coding regions | 102350 | g | - |
| CP530R | d | 125439 | 127031 | 125597 | g | a |
| NP1450L | c | 129101 | 133453 | 129488 | g | a |
| in non-coding regions | in non-coding regions | in non-coding regions | in non-coding regions | 138856 | - | a |
| D1133L | c | 140645 | 143776 | 140653 | - | t |
| I215L | c | 173844 | 174482 | 173997 | a | g |
| L11L | c | 182871 | 183152 | 183139 | a | - |

TableS1 SNP and indel information of sample 2.

| Gene name | gene locs in direct strand or complementary strand | gene start location | gene end location | site location in MK333180.1 | Nucleotides in MK333180.1 | Nucleotides in Genome of this strain |
| --- | --- | --- | --- | --- | --- | --- |
| MGF_360-2L | c | 2022 | 3110 | 2386 | a | - |
| MGF_110-3L | c | 7281 | 7655 | 7384 | c | t |
| MGF_110-7L | c | 9356 | 9769 | 9733 | c | - |
| MGF_110-14L | c | 13263 | 13613 | 13266 | c | - |
| MGF_110-13L | c | 14248 | 15072 | 14708 | - | c |
| in non-coding regions | in non-coding regions | in non-coding regions | in non-coding regions | 16664 | - | g |
| MGF_360-6L | c | 17229 | 18356 | 17455 | c | t |
| ASFV_G_ACD_00350 | d | 19002 | 19136 | 19032 | - | g |
| ASFV_G_ACD_00350 | d | 19002 | 19136 | 19032 | - | g |
| ASFV_G_ACD_00350 | d | 19002 | 19136 | 19032 | - | g |
| ASFV_G_ACD_00350 | d | 19002 | 19136 | 19032 | - | g |
| in non-coding regions | in non-coding regions | in non-coding regions | in non-coding regions | 20554 | c | t |
| in non-coding regions | in non-coding regions | in non-coding regions | in non-coding regions | 20835 | g | - |
| in non-coding regions | in non-coding regions | in non-coding regions | in non-coding regions | 20836 | g | - |
| in non-coding regions | in non-coding regions | in non-coding regions | in non-coding regions | 20837 | g | - |
| in non-coding regions | in non-coding regions | in non-coding regions | in non-coding regions | 25291 | g | - |
| in non-coding regions | in non-coding regions | in non-coding regions | in non-coding regions | 25292 | t | - |
| in non-coding regions | in non-coding regions | in non-coding regions | in non-coding regions | 25293 | t | - |
| in non-coding regions | in non-coding regions | in non-coding regions | in non-coding regions | 25294 | a | - |
| in non-coding regions | in non-coding regions | in non-coding regions | in non-coding regions | 25295 | t | - |
| in non-coding regions | in non-coding regions | in non-coding regions | in non-coding regions | 25296 | a | - |
| in non-coding regions | in non-coding regions | in non-coding regions | in non-coding regions | 25297 | a | - |
| in non-coding regions | in non-coding regions | in non-coding regions | in non-coding regions | 25298 | t | - |
| in non-coding regions | in non-coding regions | in non-coding regions | in non-coding regions | 25299 | t | - |
| in non-coding regions | in non-coding regions | in non-coding regions | in non-coding regions | 25300 | a | - |
| in non-coding regions | in non-coding regions | in non-coding regions | in non-coding regions | 25301 | c | - |
| in non-coding regions | in non-coding regions | in non-coding regions | in non-coding regions | 44265 | a | - |
| in non-coding regions | in non-coding regions | in non-coding regions | in non-coding regions | 44266 | g | - |
| in non-coding regions | in non-coding regions | in non-coding regions | in non-coding regions | 44267 | t | - |
| in non-coding regions | in non-coding regions | in non-coding regions | in non-coding regions | 44268 | t | - |
| in non-coding regions | in non-coding regions | in non-coding regions | in non-coding regions | 44269 | a | - |
| in non-coding regions | in non-coding regions | in non-coding regions | in non-coding regions | 44270 | a | - |
| in non-coding regions | in non-coding regions | in non-coding regions | in non-coding regions | 44271 | g | - |
| in non-coding regions | in non-coding regions | in non-coding regions | in non-coding regions | 44272 | a | - |
| in non-coding regions | in non-coding regions | in non-coding regions | in non-coding regions | 44273 | t | - |
| in non-coding regions | in non-coding regions | in non-coding regions | in non-coding regions | 44274 | a | - |
| in non-coding regions | in non-coding regions | in non-coding regions | in non-coding regions | 44275 | g | - |
| in non-coding regions | in non-coding regions | in non-coding regions | in non-coding regions | 44276 | t | - |
| in non-coding regions | in non-coding regions | in non-coding regions | in non-coding regions | 44277 | a | - |
| in non-coding regions | in non-coding regions | in non-coding regions | in non-coding regions | 44278 | g | - |
| in non-coding regions | in non-coding regions | in non-coding regions | in non-coding regions | 44279 | t | - |
| in non-coding regions | in non-coding regions | in non-coding regions | in non-coding regions | 44280 | t | - |
| in non-coding regions | in non-coding regions | in non-coding regions | in non-coding regions | 44281 | t | - |
| in non-coding regions | in non-coding regions | in non-coding regions | in non-coding regions | 44452 | g | - |
| in non-coding regions | in non-coding regions | in non-coding regions | in non-coding regions | 44453 | t | - |
| in non-coding regions | in non-coding regions | in non-coding regions | in non-coding regions | 44454 | t | - |
| in non-coding regions | in non-coding regions | in non-coding regions | in non-coding regions | 44455 | a | - |
| in non-coding regions | in non-coding regions | in non-coding regions | in non-coding regions | 44456 | a | - |
| in non-coding regions | in non-coding regions | in non-coding regions | in non-coding regions | 44457 | g | - |
| in non-coding regions | in non-coding regions | in non-coding regions | in non-coding regions | 44458 | t | - |
| in non-coding regions | in non-coding regions | in non-coding regions | in non-coding regions | 44459 | c | - |
| in non-coding regions | in non-coding regions | in non-coding regions | in non-coding regions | 44460 | a | - |
| in non-coding regions | in non-coding regions | in non-coding regions | in non-coding regions | 44461 | a | - |
| in non-coding regions | in non-coding regions | in non-coding regions | in non-coding regions | 44462 | t | - |
| in non-coding regions | in non-coding regions | in non-coding regions | in non-coding regions | 44463 | a | - |
| in non-coding regions | in non-coding regions | in non-coding regions | in non-coding regions | 44464 | g | - |
| in non-coding regions | in non-coding regions | in non-coding regions | in non-coding regions | 44465 | t | - |
| in non-coding regions | in non-coding regions | in non-coding regions | in non-coding regions | 44466 | t | - |
| in non-coding regions | in non-coding regions | in non-coding regions | in non-coding regions | 44467 | t | - |
| in non-coding regions | in non-coding regions | in non-coding regions | in non-coding regions | 44468 | a | - |
| in non-coding regions | in non-coding regions | in non-coding regions | in non-coding regions | 44469 | g | - |
| in non-coding regions | in non-coding regions | in non-coding regions | in non-coding regions | 44470 | t | - |
| in non-coding regions | in non-coding regions | in non-coding regions | in non-coding regions | 44471 | t | - |
| in non-coding regions | in non-coding regions | in non-coding regions | in non-coding regions | 44472 | a | - |
| in non-coding regions | in non-coding regions | in non-coding regions | in non-coding regions | 44473 | a | - |
| in non-coding regions | in non-coding regions | in non-coding regions | in non-coding regions | 44474 | g | - |
| in non-coding regions | in non-coding regions | in non-coding regions | in non-coding regions | 44475 | t | - |
| in non-coding regions | in non-coding regions | in non-coding regions | in non-coding regions | 44476 | c | - |
| in non-coding regions | in non-coding regions | in non-coding regions | in non-coding regions | 44477 | a | - |
| in non-coding regions | in non-coding regions | in non-coding regions | in non-coding regions | 44478 | a | - |
| in non-coding regions | in non-coding regions | in non-coding regions | in non-coding regions | 44479 | t | - |
| in non-coding regions | in non-coding regions | in non-coding regions | in non-coding regions | 44480 | a | - |
| in non-coding regions | in non-coding regions | in non-coding regions | in non-coding regions | 44481 | g | - |
| in non-coding regions | in non-coding regions | in non-coding regions | in non-coding regions | 44482 | t | - |
| in non-coding regions | in non-coding regions | in non-coding regions | in non-coding regions | 44483 | t | - |
| in non-coding regions | in non-coding regions | in non-coding regions | in non-coding regions | 44484 | t | - |
| in non-coding regions | in non-coding regions | in non-coding regions | in non-coding regions | 44485 | a | - |
| A240L | c | 47673 | 48383 | 47727 | c | t |
| in non-coding regions | in non-coding regions | in non-coding regions | in non-coding regions | 81729 | a | - |
| in non-coding regions | in non-coding regions | in non-coding regions | in non-coding regions | 81730 | g | - |
| in non-coding regions | in non-coding regions | in non-coding regions | in non-coding regions | 81731 | t | - |
| in non-coding regions | in non-coding regions | in non-coding regions | in non-coding regions | 81732 | g | - |
| in non-coding regions | in non-coding regions | in non-coding regions | in non-coding regions | 81733 | c | - |
| in non-coding regions | in non-coding regions | in non-coding regions | in non-coding regions | 81734 | c | - |
| in non-coding regions | in non-coding regions | in non-coding regions | in non-coding regions | 81735 | t | - |
| in non-coding regions | in non-coding regions | in non-coding regions | in non-coding regions | 81736 | g | - |
| in non-coding regions | in non-coding regions | in non-coding regions | in non-coding regions | 81737 | c | - |
| in non-coding regions | in non-coding regions | in non-coding regions | in non-coding regions | 81738 | a | - |
| in non-coding regions | in non-coding regions | in non-coding regions | in non-coding regions | 81739 | c | - |
| in non-coding regions | in non-coding regions | in non-coding regions | in non-coding regions | 81740 | a | - |
| in non-coding regions | in non-coding regions | in non-coding regions | in non-coding regions | 81741 | a | - |
| in non-coding regions | in non-coding regions | in non-coding regions | in non-coding regions | 81742 | g | - |
| in non-coding regions | in non-coding regions | in non-coding regions | in non-coding regions | 81743 | t | - |
| in non-coding regions | in non-coding regions | in non-coding regions | in non-coding regions | 81744 | g | - |
| in non-coding regions | in non-coding regions | in non-coding regions | in non-coding regions | 81745 | c | - |
| in non-coding regions | in non-coding regions | in non-coding regions | in non-coding regions | 81746 | t | - |
| in non-coding regions | in non-coding regions | in non-coding regions | in non-coding regions | 81747 | t | - |
| in non-coding regions | in non-coding regions | in non-coding regions | in non-coding regions | 81748 | g | - |
| in non-coding regions | in non-coding regions | in non-coding regions | in non-coding regions | 81749 | c | - |
| in non-coding regions | in non-coding regions | in non-coding regions | in non-coding regions | 81750 | a | - |
| in non-coding regions | in non-coding regions | in non-coding regions | in non-coding regions | 81751 | c | - |
| in non-coding regions | in non-coding regions | in non-coding regions | in non-coding regions | 81752 | a | - |
| in non-coding regions | in non-coding regions | in non-coding regions | in non-coding regions | 81753 | a | - |
| in non-coding regions | in non-coding regions | in non-coding regions | in non-coding regions | 81754 | g | - |
| in non-coding regions | in non-coding regions | in non-coding regions | in non-coding regions | 81755 | t | - |
| in non-coding regions | in non-coding regions | in non-coding regions | in non-coding regions | 81756 | g | - |
| in non-coding regions | in non-coding regions | in non-coding regions | in non-coding regions | 81757 | c | - |
| in non-coding regions | in non-coding regions | in non-coding regions | in non-coding regions | 81758 | c | - |
| in non-coding regions | in non-coding regions | in non-coding regions | in non-coding regions | 81759 | t | - |
| in non-coding regions | in non-coding regions | in non-coding regions | in non-coding regions | 81760 | g | - |
| in non-coding regions | in non-coding regions | in non-coding regions | in non-coding regions | 81761 | c | - |
| in non-coding regions | in non-coding regions | in non-coding regions | in non-coding regions | 81762 | a | - |
| in non-coding regions | in non-coding regions | in non-coding regions | in non-coding regions | 81763 | c | - |
| in non-coding regions | in non-coding regions | in non-coding regions | in non-coding regions | 81764 | a | - |
| in non-coding regions | in non-coding regions | in non-coding regions | in non-coding regions | 81765 | a | - |
| in non-coding regions | in non-coding regions | in non-coding regions | in non-coding regions | 81766 | g | - |
| in non-coding regions | in non-coding regions | in non-coding regions | in non-coding regions | 81767 | t | - |
| in non-coding regions | in non-coding regions | in non-coding regions | in non-coding regions | 81768 | g | - |
| in non-coding regions | in non-coding regions | in non-coding regions | in non-coding regions | 81769 | c | - |
| in non-coding regions | in non-coding regions | in non-coding regions | in non-coding regions | 81770 | t | - |
| in non-coding regions | in non-coding regions | in non-coding regions | in non-coding regions | 81771 | t | - |
| in non-coding regions | in non-coding regions | in non-coding regions | in non-coding regions | 81772 | g | - |
| in non-coding regions | in non-coding regions | in non-coding regions | in non-coding regions | 81773 | c | - |
| in non-coding regions | in non-coding regions | in non-coding regions | in non-coding regions | 81774 | a | - |
| in non-coding regions | in non-coding regions | in non-coding regions | in non-coding regions | 81775 | c | - |
| in non-coding regions | in non-coding regions | in non-coding regions | in non-coding regions | 81776 | a | - |
| in non-coding regions | in non-coding regions | in non-coding regions | in non-coding regions | 81777 | a | - |
| in non-coding regions | in non-coding regions | in non-coding regions | in non-coding regions | 81778 | g | - |
| in non-coding regions | in non-coding regions | in non-coding regions | in non-coding regions | 81779 | t | - |
| in non-coding regions | in non-coding regions | in non-coding regions | in non-coding regions | 81780 | g | - |
| in non-coding regions | in non-coding regions | in non-coding regions | in non-coding regions | 81781 | c | - |
| in non-coding regions | in non-coding regions | in non-coding regions | in non-coding regions | 81782 | t | - |
| in non-coding regions | in non-coding regions | in non-coding regions | in non-coding regions | 81783 | t | - |
| in non-coding regions | in non-coding regions | in non-coding regions | in non-coding regions | 81784 | g | - |
| in non-coding regions | in non-coding regions | in non-coding regions | in non-coding regions | 81785 | c | - |
| in non-coding regions | in non-coding regions | in non-coding regions | in non-coding regions | 81786 | a | - |
| in non-coding regions | in non-coding regions | in non-coding regions | in non-coding regions | 81787 | c | - |
| in non-coding regions | in non-coding regions | in non-coding regions | in non-coding regions | 81788 | a | - |
| in non-coding regions | in non-coding regions | in non-coding regions | in non-coding regions | 81789 | a | - |
| in non-coding regions | in non-coding regions | in non-coding regions | in non-coding regions | 81790 | g | - |
| in non-coding regions | in non-coding regions | in non-coding regions | in non-coding regions | 81791 | t | - |
| in non-coding regions | in non-coding regions | in non-coding regions | in non-coding regions | 81792 | g | - |
| in non-coding regions | in non-coding regions | in non-coding regions | in non-coding regions | 81793 | c | - |
| in non-coding regions | in non-coding regions | in non-coding regions | in non-coding regions | 81794 | t | - |
| in non-coding regions | in non-coding regions | in non-coding regions | in non-coding regions | 81795 | t | - |
| in non-coding regions | in non-coding regions | in non-coding regions | in non-coding regions | 81796 | a | - |
| in non-coding regions | in non-coding regions | in non-coding regions | in non-coding regions | 81797 | c | - |
| in non-coding regions | in non-coding regions | in non-coding regions | in non-coding regions | 81798 | a | - |
| in non-coding regions | in non-coding regions | in non-coding regions | in non-coding regions | 81799 | c | - |
| in non-coding regions | in non-coding regions | in non-coding regions | in non-coding regions | 81800 | a | - |
| C475L | c | 85734 | 87161 | 85894 | g | a |
| D1133L | c | 140645 | 143776 | 140653 | - | t |

TableS2 SNP and indel information of sample 1.

| TGS assamble location | NGS assamble location | nucleotide in this location of TGS assamble | nucleotide in this location of NGS assamble |
| --- | --- | --- | --- |
| 6941 | 6942 | - | a |
| 12986 | 12986 | - | c |
| 12987 | 12986 | - | c |
| 12988 | 12986 | - | c |
| 12989 | 12986 | - | c |
| 14429 | 14425 | - | c |
| 14430 | 14425 | - | c |
| 14431 | 14425 | - | c |
| 14432 | 14425 | - | c |
| 14433 | 14425 | - | c |
| 14434 | 14425 | - | c |
| 14435 | 14425 | - | c |
| 14436 | 14425 | - | c |
| 14437 | 14425 | - | c |
| 14438 | 14425 | - | c |
| 14439 | 14425 | - | c |
| 14440 | 14425 | - | c |
| 14441 | 14425 | - | c |
| 14442 | 14425 | - | c |
| 14443 | 14425 | - | c |
| 14444 | 14425 | - | c |
| 14445 | 14425 | - | c |
| 14446 | 14425 | - | c |
| 14447 | 14425 | - | c |
| 14448 | 14425 | - | c |
| 14449 | 14425 | - | c |
| 17882 | 17857 | - | t |
| 18569 | 18543 | g | - |
| 18777 | 18751 | - | g |
| 18778 | 18751 | - | g |
| 18779 | 18751 | - | g |
| 18780 | 18751 | - | g |
| 18781 | 18751 | - | g |
| 18782 | 18751 | - | g |
| 18783 | 18751 | - | g |
| 18784 | 18751 | - | g |
| 18785 | 18751 | - | g |
| 20575 | 20540 | - | g |
| 20576 | 20540 | - | g |
| 26205 | 26168 | t | - |
| 36570 | 36533 | a | - |
| 44009 | 43972 | - | t |
| 44010 | 43972 | - | a |
| 44011 | 43972 | - | a |
| 44012 | 43972 | - | g |
| 44013 | 43972 | - | a |
| 44014 | 43972 | - | t |
| 44015 | 43972 | - | a |
| 44016 | 43972 | - | g |
| 44017 | 43972 | - | t |
| 44018 | 43972 | - | a |
| 44019 | 43972 | - | g |
| 44020 | 43972 | - | t |
| 44021 | 43972 | - | t |
| 44022 | 43972 | - | t |
| 44023 | 43972 | - | a |
| 44024 | 43972 | - | g |
| 44025 | 43972 | - | t |
| 44177 | 44123 | - | t |
| 44178 | 44123 | - | t |
| 44179 | 44123 | - | a |
| 44180 | 44123 | - | a |
| 44181 | 44123 | - | g |
| 44182 | 44123 | - | t |
| 44183 | 44123 | - | c |
| 44184 | 44123 | - | a |
| 44185 | 44123 | - | a |
| 44186 | 44123 | - | t |
| 44187 | 44123 | - | a |
| 44188 | 44123 | - | g |
| 44189 | 44123 | - | t |
| 44190 | 44123 | - | t |
| 44191 | 44123 | - | t |
| 44192 | 44123 | - | a |
| 44193 | 44123 | - | g |
| 44194 | 44123 | - | t |
| 44195 | 44123 | - | t |
| 44196 | 44123 | - | a |
| 44197 | 44123 | - | a |
| 44198 | 44123 | - | g |
| 44199 | 44123 | - | t |
| 44200 | 44123 | - | c |
| 44201 | 44123 | - | a |
| 44202 | 44123 | - | a |
| 44203 | 44123 | - | t |
| 44204 | 44123 | - | a |
| 44205 | 44123 | - | g |
| 44206 | 44123 | - | t |
| 44207 | 44123 | - | t |
| 44208 | 44123 | - | t |
| 44209 | 44123 | - | a |
| 44210 | 44123 | - | g |
| 59199 | 59111 | t | - |
| 72044 | 71956 | - | t |
| 75078 | 74989 | t | - |
| 78921 | 78832 | t | - |
| 81285 | 81196 | t | - |
| 111495 | 111406 | - | a |
| 116237 | 116147 | a | - |
| 128131 | 128041 | t | - |
| 128245 | 128155 | - | t |
| 135582 | 135491 | a | - |
| 138824 | 138733 | t | - |
| 146567 | 146476 | - | a |
| 166757 | 166665 | - | t |
| 167545 | 167452 | a | - |
| 168345 | 168252 | a | - |
| 173416 | 173323 | - | t |
| 183111 | 183017 | - | t |
| 186431 | 186336 | a | - |

TableS3. Different site between NGS and TGS assamble.
